# Catalytic hydrotreating of bio-oil and evaluation of main noxious emissions of gaseous phase

**DOI:** 10.1038/s41598-021-85244-z

**Published:** 2021-03-17

**Authors:** Rami Doukeh, Dorin Bombos, Mihaela Bombos, Elena-Emilia Oprescu, Gheorghe Dumitrascu, Gabriel Vasilievici, Catalina Calin

**Affiliations:** 1 Chemistry Department, Petroleum-Gas University of Ploiesti, 39 Bucuresti Blvd., 100680 Ploiesti, Romania; 2 National Institute for Research and Development for Chemistry and Petrochemistry ICECHIM BucurestiNational Institute for Research and Development for Chemistry and Petrochemistry ICECHIM Bucuresti, 202 Splaiul Independentei, 060021 Bucharest, Romania; 3 Department of Engineering Thermodynamics, ‘‘Gh. Asachi’’ Technical University of Iasi, Bd. D. Mangeron, 59-61, 6600 Iasi, Romania

**Keywords:** Biofuels, Chemical engineering

## Abstract

Bio-oil produced from biomass pyrolysis has the potential to become an alternative renewable fuel. However due to the high content of oxygenated compounds is unsuitable as transportation fuel. The objective of this work is to evaluate the catalytic activity of CoMo /γ-Al_2_O_3_-HMS in the hydrotreating process of biomass pyrolysis bio-oil. The prepared catalyst was characterized by different techniques (X-ray diffraction (XRD), transmission electron microscopy (TEM), Fourier transform infrared spectroscopy (FT-IR) and X-ray photoelectron spectroscopy (XPS)) analysis. The experiments were carried out in a flow fixed-bed reactor at the temperature range of 250–320 °C, pressure between 20–40 bar, and LHSV of 3 h^-1^. The results showed that at mild conditions of 320 °C and 40 bar, the catalyst is very active in the hydrotreating process leading to a decrease of total acid number of hydrotreated bio-oil with almost 89% and bio-oil conversion of 87.23%. In addition, in order to evaluate the harmful emissions resulted from combustion of gaseous phase obtained in the hydrotreating process a chemical modelling algorithm was developed.

## Introduction

Biogas plants are alternative sources for renewable energy, biomass waste treatment and organic fertilizers (digestion waste, i.e. digestate). However, an accumulation of biogas plants in certain regions might lead to an oversupply of digestate^1^, causing many environmental concerns (odor control, transportation cost, pathogen, heavy metal (loid) contamination)^2^. Digestate pyrolysis can be an environmentally and energy recovery solution for digestate disposal^3^.Typically, this process is performed between 400 and 700 °C at near atmospheric pressure or below, in the absence of oxygen. Bio-oil produced from biomass pyrolysis has the potential to become an alternative renewable fuel and raw material for fine chemicals production generating thus bio-based high added value products^4^.

In general, pyrolysis oil contains hundreds of organic compounds including hydrocarbons and oxygenated compounds (i.e. organic acids, aldehydes, ketones and phenolics)^5^. The presents of these compounds cause bio-oil to have low heating value, low solubility in fuels such as diesel/gasoline, poor thermal and chemical stability and high acidity, high viscosity and high corrosiveness than petroleum^6,7^. However, pyrolysis oil can be converted to transportation fuel by catalytic treatment. The catalysts and conditions used are very similar to those used in petroleum hydrodesulfurization, hydrotreating, and hydrocracking processes, more generally described as hydroprocessing^8^. A promising upgrading technology is considered to be catalytic hydrotreatment of bio-oil, which involves treatment of pyrolysis oil with hydrogen in the presence of a heterogeneous catalyst leading to gasoline or diesel like products^9^.

Different hydrotreating catalysts have been investigated, such as Ni/SiO_2_, Ni/ZrO_2_, Ni/CeO_2_, Ni/Al_2_O_3_, Ni-Cu/SiO_2_, Ru/C, Pt/C, Pd/C, Pd/SiO_2_, Pt-Ni/SiO_2_, Pd–Ni/SiO_2_ and Cu/SBA-15^10^. Wildschut J. et al.,^11^ compared the results of typical hydrotreatment catalysts (sulfide NiMo/Al_2_O_3_ and CoMo/Al_2_O_3_) with heterogeneous noble-metal catalysts (Ru/C, Ru/TiO_2_, Ru/Al_2_O_3_, Pt/C, and Pd/C). The Ru/C catalyst was found to be superior to the classical hydrotreating catalysts with respect to oil yield (up to 60 wt %) and deoxygenation level (up to 90 wt %). However, due to the high cost and low availability of noble metals, their industrial applications are more difficult^12^. Moreover, efforts on increasing the economic performance of bio-oil hydrotreating technology lies on the application of non-noble metal catalysts, mild reaction condition (low temperature and hydrogen pressure) and high selectivity towards desired products. Nevertheless, the design of highly active and stable catalysts remains as the key challenge which plays a significant role in the implementation of biomass in the future biorefinery schemes^13^. In recent years, non-noble metal catalysts based on less-expensive metals such as Ni, Co and Mo have been reported to exhibit high activity and selectivity for decarboxylation/decarbonylation process^14^. Among them, NiMo and CoMo sulfides catalyst have been widely used in bio-oil hydrotreating. However, the main disadvantage of these catalysts is the requirement of the presence of sulfur in the processed feeds to maintain their activity and selectivity and, hence, end products is inevitably contaminated by sulfur^13,15^. Also, sulphided catalysts are rapidly deactivated by water^9^ and coke formation^16^. In order to solve these issues, non-sulfided NiMo or MoCo catalysts on various supports have been studied^17^. However, many challenges remain to improve the catalyst activity, stability, and selectivity at minimum amounts of hidrogen (i.e., low hidrogen pressures), and low temperatures, because high hydrogen pressure and temperatures, favor coke formation and thus leading to reactor fouling and product deterioration^18,19^. Significant amounts of coke could form during the hydrotreating at elevated temperature due to the insufficient active hydrogen supply, which cannot match the demand of the broken bonds. Furthermore, coke formation is a complicated process that is poorly studied^20^. Although the commercial CoMo and NiMo catalysts are commonly used at high temperatures of above 350 °C, they are sufficiently active at lower temperatures^15,17,21^. Nevertheless, the information regarding the application of non-noble catalysts such as CoMo at low temperatures and pressure is rather limited^15^. In this context, the objective of this paper is to study, the catalytic upgrading of digestat pyrolysis oil via mild hydrotreating process of over CoMo /γ-Al_2_O_3_-HMS. The influence of temperature and pressure on bio-oil conversion and deoxygenation efficiency was investigated. In addition, a chemical modelling algorithm was developed in order to evaluate the harmful emissions resulted from combustion of hydrotreating gaseous phase. This calculation algorithm can be applied to any pressurized or atmospheric combustion engine fueled with different mixture of combustible gases such as: natural gas, hydrogen-enriched natural gas and oxy-fuel.

## Results and discussion

### Catalyst characterization

The textural properties, such as specific surface area, pore size distribution and pore volume of the γ-Al_2_O_3_-HMS support and corresponding CoMo catalyst, were evaluated by nitrogen adsorption–desorption isotherms using Brunauer-Emmet-Teller (BET) method (Table 1). The γ-Al_2_O_3_-HMS support has a high specific surface area of 308.37 m^2^/g, a mean pore diameter of 5.49 nm and a total pore volume of 0.631 cm^3^/g. The addition of Co and Mo to γ-Al_2_O_3_-HMS support leads to a decrease in textural properties. The pore size and pore volume reflect a decrease of about 30% after impregnation indicating that impregnation may block the pores (Table 1).

**Table 1:** Textural properties of the support and of the catalyst.

Sample	Surface area (m^2^/g)	Pore volume (cc/g)	Mean pore diameter Dv(d) (nm)
γ-Al_2_O_3_-HMS	308.37	0.631	5.49
CoMo/γ-Al_2_O_3_-HMS	280	0.440	3.66

**Figure 1:**
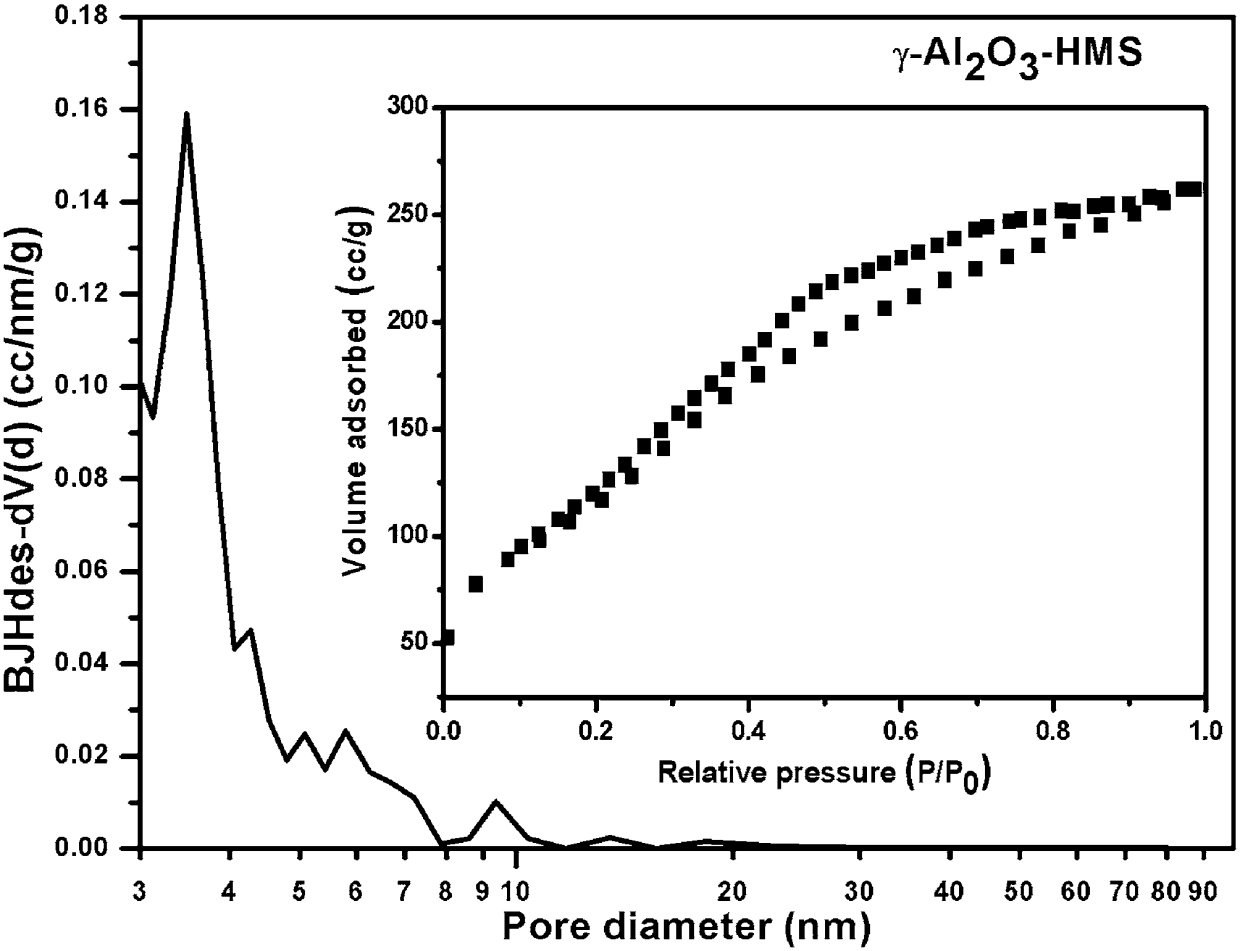
Pore size distribution and adsorption–desorption isotherm of the γ-Al_2_O_3_ –HMS support.

**Figure 2:**
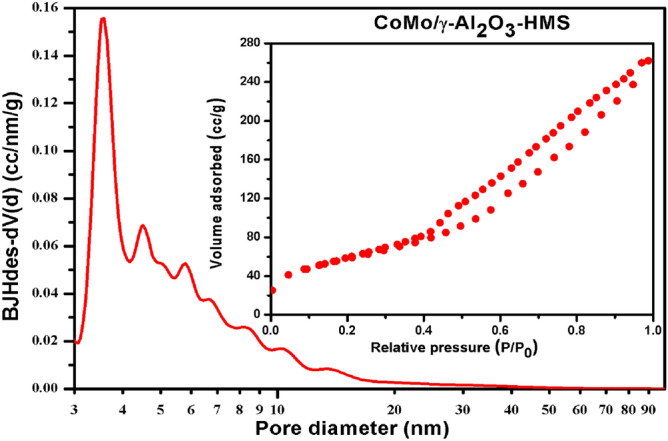
Pore size distribution and adsorption–desorption isotherm of the CoMo/γ-Al_2_O_3_ –HMS catalyst.

The N_2_ adsorption–desorption isotherm of the support and its catalyst is attributed to IV type isotherm (Figs. 1, 2) and the nitrogen isotherm reveals a hysteresis loop of H2 and H3-type respectively. The alteration of hysteresis loop shape for CoMo/γ-Al_2_O_3_-HMS catalyst also indicate that there is plugging of mesopores and there are changes in the support structure after impregnation.

**Figure 3:**
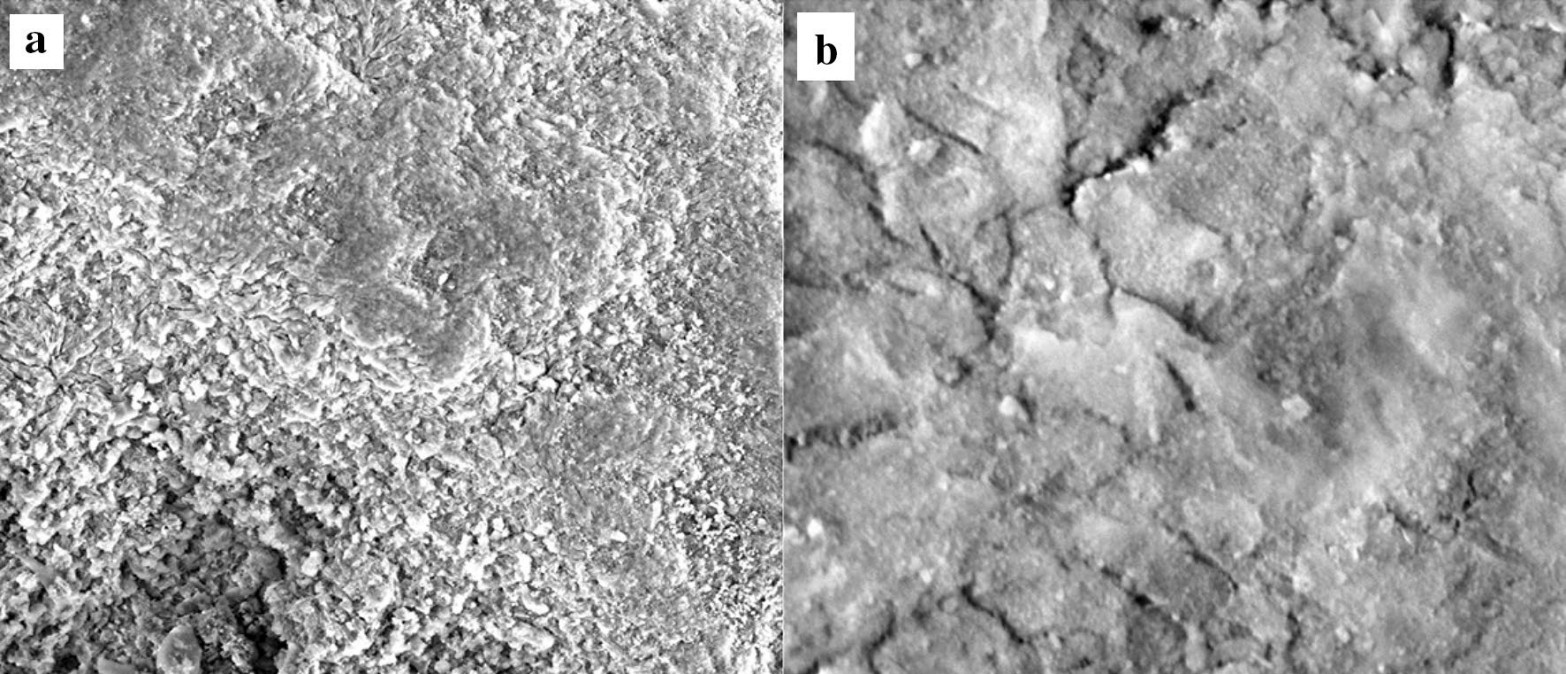
SEM images of the catalyst CoMo/γ-Al_2_O_3_-HMS **a**100 µm and **b** 5 µm image scale bar.

Scanning Electron Microscopy (SEM) images of the synthesized CoMo/γ-Al_2_O_3_-HMS catalyst are shown in Fig. 3 with different magnifications (the left-side image scale bar is 100 µm and the right-side 5 µm) and show the homogeneity of the sample.

**Figure 4:**
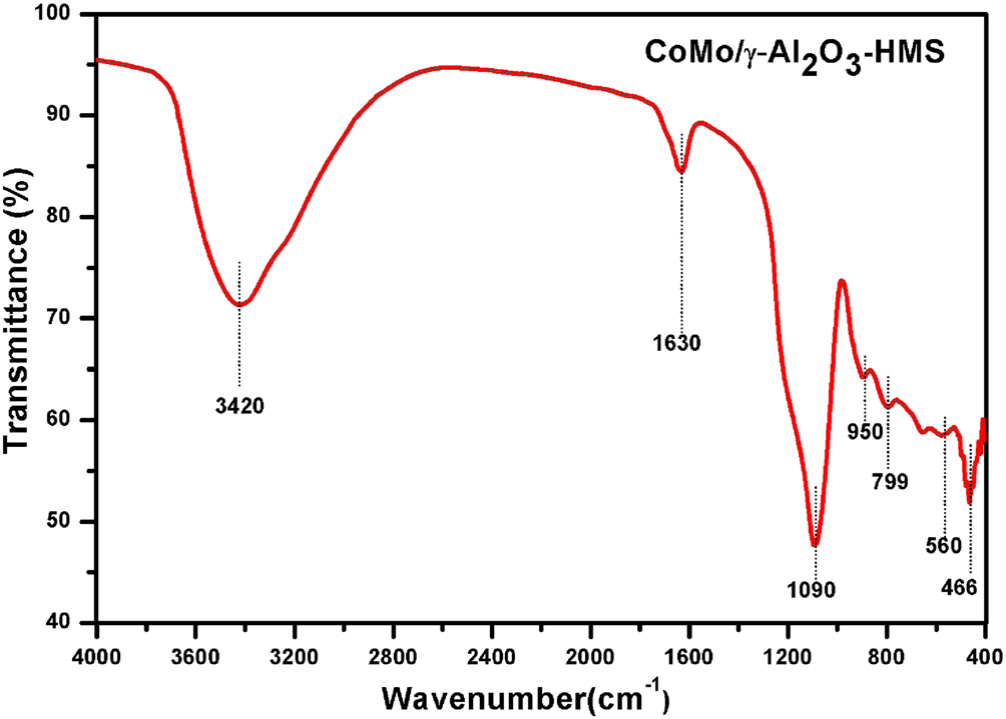
IR spectrum of CoMo/γ-Al_2_O_3_-HMS catalyst.

The Fourier transform infrared (FT-IR) spectrum of the catalyst (Fig. 4) shows broad bands around 3420 cm^-1^ and 1630 cm^-1^, which were assigned to stretching and bending modes of –OH group of water molecules on the surface of the solid^22^. The peaks in the region of 450–800 cm^-1^ were assigned to the oxide structures. The accurate assignment of the bands to specific compounds was difficult because the different compound bands overlap. The Si–O–Si specific bands (from HMS) are assigned at 1090 cm^-1^, 950 and 800 cm^−1^^23^.

**Figure 5:**
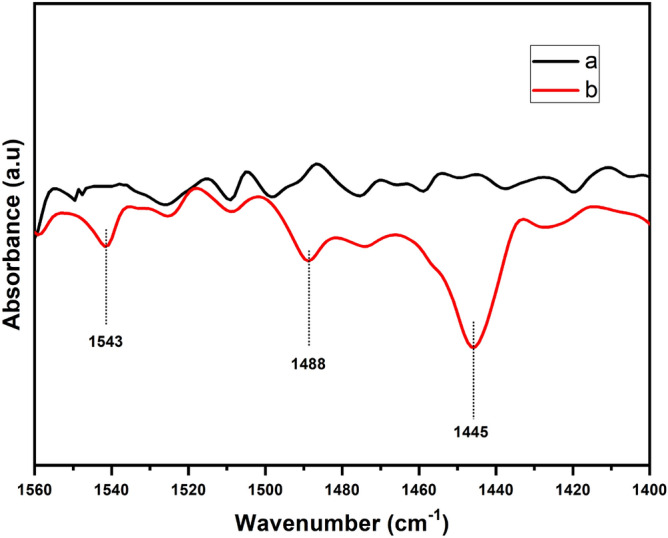
Pyridine-adsorbed FT-IR spectrum of CoMo/γ-Al_2_O_3_-HMS catalyst.

The acidic properties of CoMo/γ-Al_2_O_3_-HMS catalys were evaluated through the FT-IR analysis of pyridine adsorption (Fig. 5). The pyridine-adsorbed FT-IR spectrum showed features in the region of 1400–1600 cm^−1^ due to the stretching vibrations of M–N (metal–nitrogen) and N–H (pyridinium ion). The band assigned to pyridine adsorbed onto Lewis-type acid sites is recorded at 1445 cm^−1^, the band at 1543 cm^−1^ is due to pyridine adsorbed on Brönsted-type acid sites and the band around wavenumber 1488 cm^−1^ is due to physisorbed pyridine^24,25^. The calculated results are 97.41 μmol/cm for Lewis-type acid sites and 18.83 μmol/cm for Brönsted-type acid sites.The acid site density of 1.64 × 10^18^ sites/m^2^ and total acidity of 4.59 × 10^20^ sites/g of the catalyst were calculated according to A.I. Osman et al.^26^ (Figure S1).

**Figure 6:**
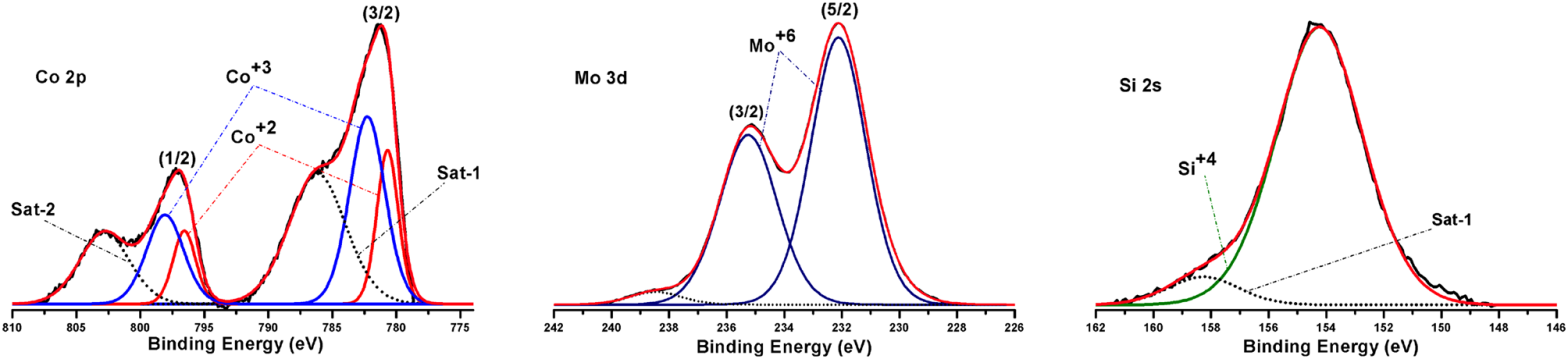
XPS Co 2p, Mo 3d and Si 2 s spectra of CoMo/γ-Al_2_O_3_-HMS catalyst.

**Figure 7:**
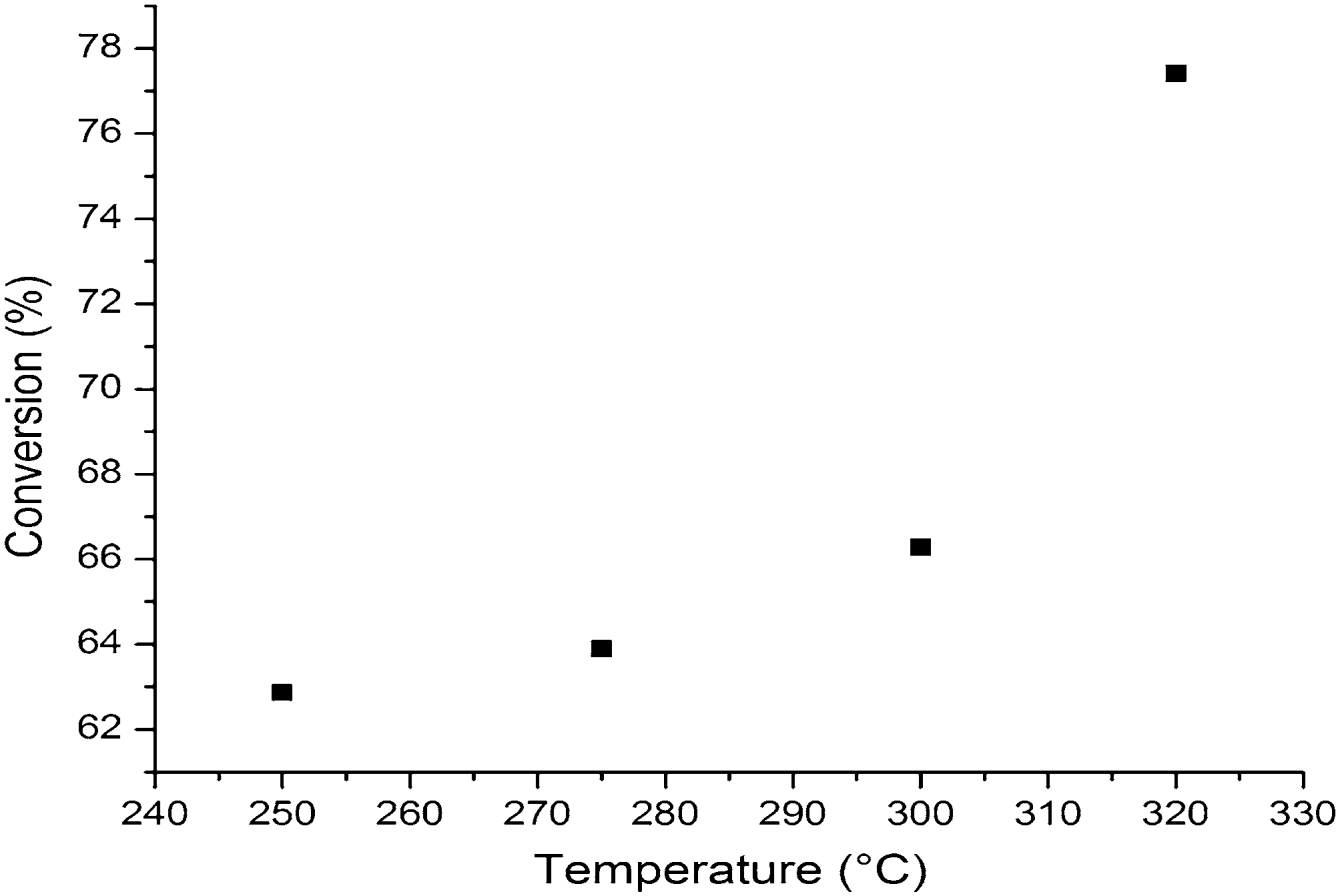
The influence of temperature over bio-oil conversion.

The surface components and chemical states of metal species on the catalysts were studied by X-Ray Photoelectron Spectroscopy (XPS) method. Results obtained by this technique are shown in Fig. 7 and Table 2. XPS signals were assigned according to the literature and the official web page of National Institute of Standards and Technology (NIST)^27^. Binding energies in the ranges of 780–783 eV and 232 eV are ascribed to Co2p and Mo3d spectra and 154 eV to Si 2 s. The characteristic Mo 3d_5/2_ peak at 232.12 eV indicate the presence of Mo^6+^ which can be attributed to MoO_3_^28,29^. Co is present as Co^3+^ and Co^2+^ species. The binding energy of Co^3+^, with the Co 2p_3/2_ profile, is 782.27 eV which could be ascribed to Co_3_O_4_ and CoOOH^30,31^ Co^2+^ may be present as CoO and CoAl_2_O_4_^32^ with Co 2p_3/2_ peak at 780.68 eV. From the calcination step of the catalyst preparation it could also be formed Co and Mo aluminates or double oxides containing Co and Mo. According to Fig. 6 and Table 2, Si (from HMS mesoporous silica) occurs in the Si 2 s energy range of 154.23 eV, assigned to Si^4+^ (SiO_2_).

**Table 2:** XPS binding energies and assigned species for Co 2p, Mo 3d and Si 2 s.

Element	Spectral line	Energy (eV)	Assigned species
Co	2p_3/2_	782.27	Co^+3^
	2p_3/2_	780.68	Co^+2^
Mo	3d_5/2_	232.12	Mo^+6^
Si	2 s	154.23	Si^4+^

### Chemical composition of pyrolysis bio-oil

The chemical composition of pyrolysis bio-oil is shown in Table S1. The main compounds identified were unsaturated organic compounds, carbonyl compounds, furan derivatives, phenols, lower carboxylic acids and unsaturated fatty acids. These compounds have a different polarity and a wide range of boiling point. Compounds with high reactivity containing olefin groups conjugated with carbonyl groups (i.e. cyclopenten-1-one derivatives) were also been identified. These compounds can deactivate the catalyst at high temperatures due to the formation of oligomers.

The physical characteristics of the pyrolysis bio-oil (Table 3) show relatively high water content (over 25 w.t % ), density about 0.9956 g / mL due to the presence of water and short carboxylic acids (acetic, propionic and butyric ) in relatively high concentrations. The presence of short carboxylic acids and fatty acids justifies the high total acid number and saponification number of almost 135 and respectively, 224.12 mg KOH /g.

**Table 3:** The physical characteristics of the pyrolysis bio-oil.

Property	Value	UM
Density	0.9956	g/mL
Water content	25.10	%
Total acid number	134.9	mg KOH/g
Saponification number	224.12	mg KOH/g

### Catalysts activity evaluation

The catalytic activity of CoMo /γ-Al_2_O_3_-HMS for the bio-oil hydrotreating was evaluated in the temperature range of 250–320 °C, pressure between 20–40 bar, and constant LHSV of 3 h^−1^.

The effect of temperature over bio-oil yield is presented in Fig. 7. The results showed that the bio-oil conversion increases gradually with temperatures, from 62.86% to over 77.42% at maxim temperature of 320 °C. This behavior is due to the different reactivity of the various classes of oxygenated compounds present in the bio-oil. The phenolic as well as the carbonyl compounds are reactive in the hydrotreating process from lower temperature values of 250 °C. Meanwhile, the carboxylic compounds have a lower reactivity in the deoxygenation at temperature value of 250 °C, thus the hydrotreating process of these compounds begins at temperatures above 300 °C.

**Figure 8:**
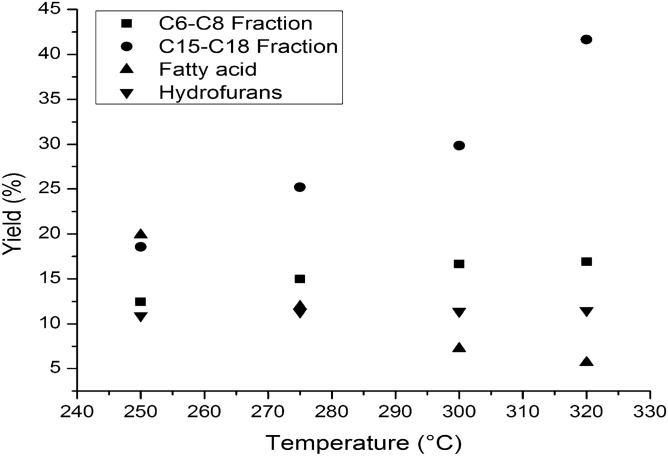
The influence of temperature over the yields in products composition of liquid organic phase.

Figure 8 presents the yields of products composition of liquid organic phase resulted after bio-oil hydrotreating process. The yields of C_6_-C_8_ hydrocarbon and furan fractions have similar values at temperatures up to 275 °C, after which the yield of furans decreased slightly with temperature reaching about 10% at 320 °C, due to their lower reactivity in the hydrodeoxygenation process. The yield of fatty acid composition decreased with increasing temperature reaching the lowest value about 5% at 320 °C. The yield of C_15_-C_18_ hydrocarbon fraction increases with increasing temperature, perhaps due to the hydro-decarboxylation reaction of the fatty acids from the waste vegetable oil fraction. As expected, the total acid number of hydrotreated bio-oil decreases with temperature with almost 89%, from 135.9 to almost 15 mg KOH/g.

**Figure 9:**
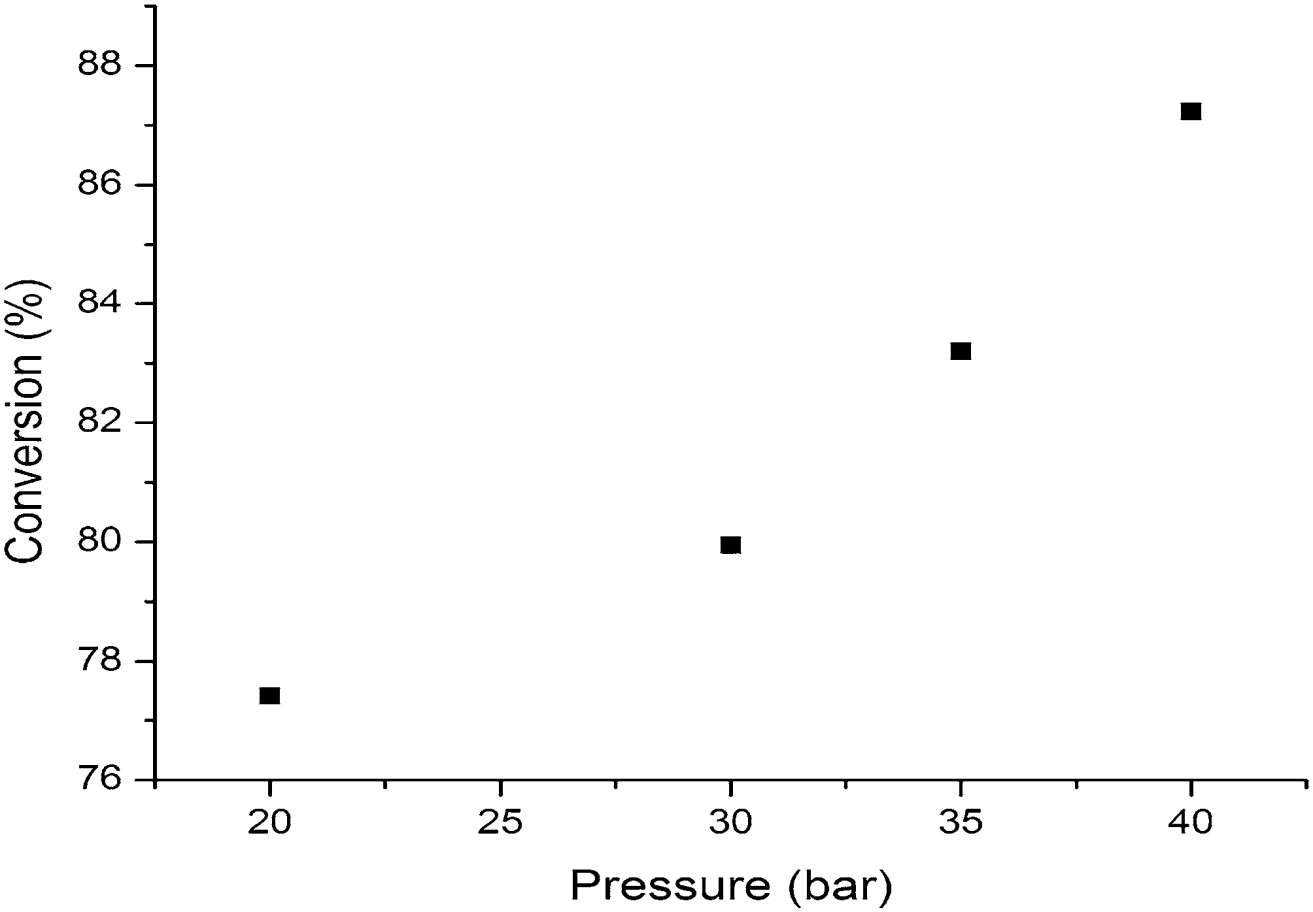
The influence of temperature over bio-oil conversion.

Minimizing the consumption of hydrogen is an important issue for hydrotreating process of bio-oil due to the possibility of obtaining ring saturated products via unwanted hydrogenation process under high hydrogen pressure. These compounds decrease the octane number of the upgraded product which could hinder its direct utilization as fuel or blending with conventional oil or in the forthcoming bio-refineries^33^. The latest results of Mo-based catalysts show great potential for converting lignin-derived bio-oil into aromatic hydrocarbons at low hydrogen pressure^13^. In this regard, we study the influence of pressure over bio-oil conversion during hydrotrating process in the range of 20–40 bar at constant temperature of 320 °C. As can be observed in Fig. 9, the conversion of bio-oil increases with pressure, the increase being more pronounced in the range of 30–40 bar. It is observed that low pressures (20–30 bar) favors preferentially the hydrodeoxygenation reaction of carbonyl and phenolic compounds^34,35^ while higher pressures hydrodeoxygenate the carboxylic compounds^21,36^. Therefore, at 20 bar the bio-oil conversion was of 77.42 and increased to 87.23% at 40 bar.

**Figure 10:**
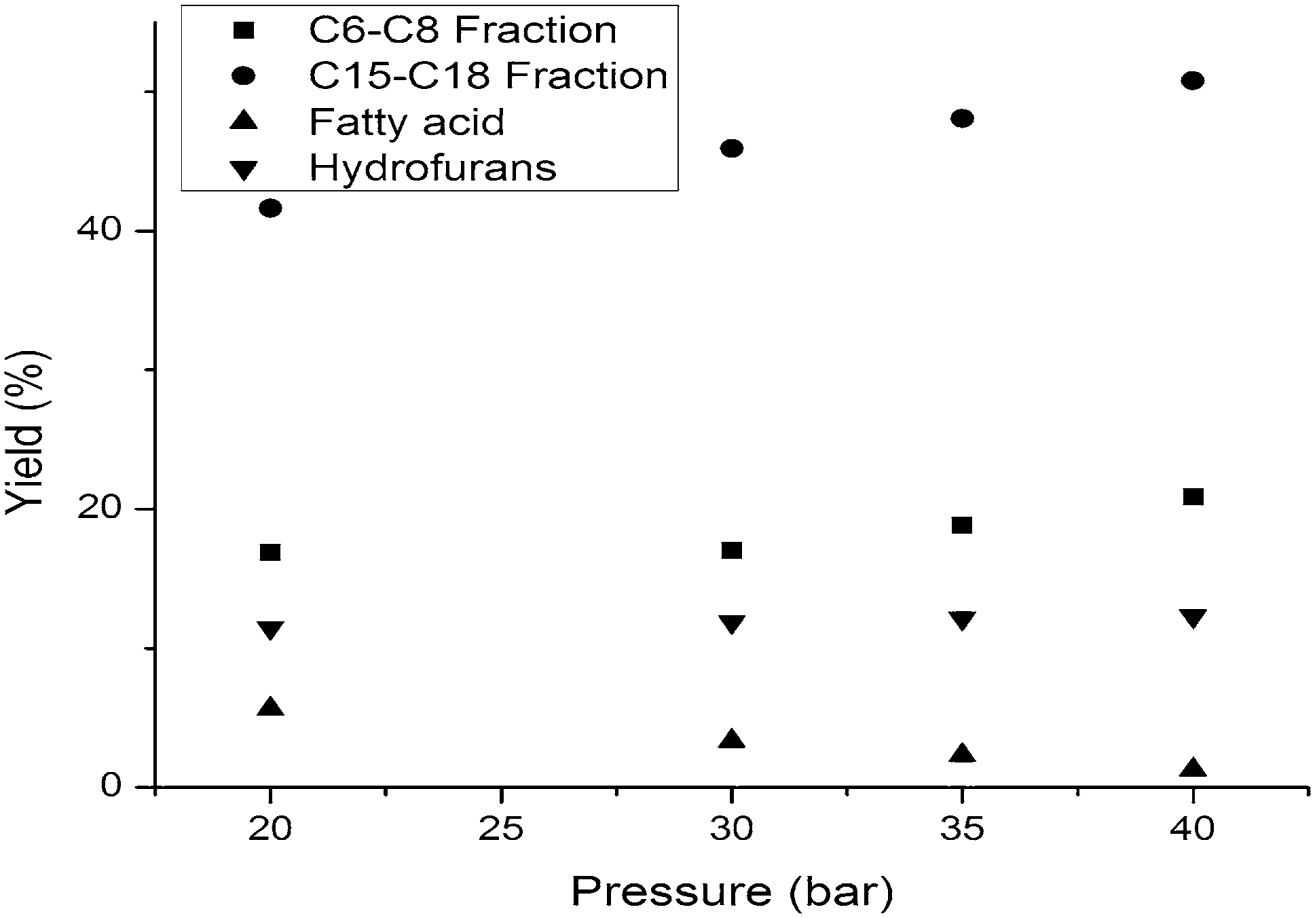
The influence of temperature over the yields in products composition of liquid organic phase.

Regarding the yields of products composition, high pressure favors the decarboxylation of the lipids and fatty acids, so that the yield of the C_15-_C_18_ hydrocarbon fraction increases from 41.64 at 20 bar to maximum value of 50.79 at 40 bar. In contrast, the yield of C_6_-C_8_ hydrocarbon fraction, resulting mainly from the hydrotreating of phenols and carbonyl compounds, is not significantly influenced by the pressure variation, the growth being insignificantly higher. The yield of furans does not change practically on the studied pressure range, the efficiency of the catalyst studied in the hydrotreating process of furans being relatively low at these pressure values (Fig. 10). The total acid number of the hydrotreated bio-oil decreases with the increase of the pressure, behavior explained by the increase activity of the Co-Mo catalyst, at pressure over 30 bar, for hydro-decarboxylation process.

### Evaluation of main noxious emissions of bio-oil hydrotreating gaseous phase

After each experiment, the gas-phase composition was analyzed, and the most representative data are given in Table 4. The main component of gaseous phase is unreacted hydrogen, followed by CO and C_1_-C_4_ hydrocarbons in different percent depending on the experimental parameters used.

**Table 4:** The mole composition in the flow section “0”.

Fuel N_f_ = 1 kmole								Air	
n_H2_ [kmole]	n_CO_ [kmole]	n_CH4_ [kmole]	n_C2H4_ [kmole]	n_C2H6_ [kmole]	n_C3H6_ [kmole]	n_C3H8_ [kmole]	n_C4H10_ [kmole]	n_O2_ [kmole]	n_N2_ [kmole]
0.27867	0.161	0.45878	0.03589	0.01879	0.01335	0.006222	0.0273	(1 + x)^.^n_O2,min_	(1 + x)^.^n_N2,min_

where n_O2,min_ = 0.5 n_H2_ + 0.5 n_CO_ + 2 n_CH4_ + 3 n_C2H4_ + 3.5 n_C2H6_ + 4.5 n_C3H6_ + 5 n_C3H8_ + 6.5 n_C4H10_ = 1.5795 kmole,

n_N2,min_ = 3.7619^.^n_O2,min_ = 5.9418 kmole and.

x is the excess air/oxygen.

Unlike natural gas which has been intensively studied in the combustion process, synthesis gas fuel mixtures have not been widely investigated. Therefore, an evaluation of main noxious emissions was performed through a pure chemical modeling algorithm developed. This chemical modeling algorithm allows to chemically analyze the constant pressure combustion for any gaseous mixture fuels. The chemical model chose the minimum and necessary chemical equations in order to quantify the air based combustion noxious.

The evaluation of main noxious emissions was performed through a pure chemical modeling algorithm developed by authors at necessity. This chemical modeling algorithm allows to chemically analyze the constant pressure combustion for any gaseous mixture fuels. The chemical model chose the minimum and necessary chemical equations in order to quantify the air based combustion noxious. The main combustion noxious emissions, i.e. CO_2_, CO and NOx, for the gaseous mixture fuel were evaluated through a combustion chemistry model involving the energy and mass balance equations applied to three types of chemical reactions:

- primary oxidation of inlet chemical species of gaseous fuel,
- secondary dissociation chemical reactions and,
- tertiary recombination chemical reactions.

**Figure 11:**
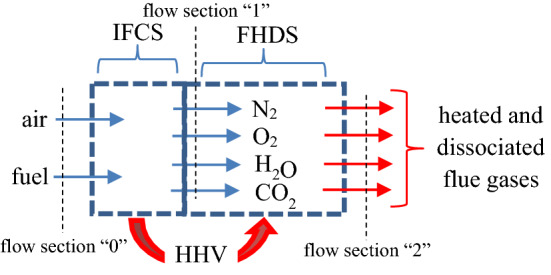
The scheme of the combustion process.

The combustion was thought to be made in two successive fictitious steps complying with energy and mass balance laws, see Fig. 11.

- the first step is an isothermal and constant pressure combustion at the standard temperature T_0_ = 298 K and at an imposed pressure p ≥ p_0_, with p_0_ = 0.1 MPa; this step is performed inside an isothermal fictitious combustion space, IFCS, and it is conceived without dissociation;
- the second step is heating with dissociation of flue gases produced in the first step; this process takes place inside the second fictitious heating and dissociation space, FHDS, and here it is consuming the heat released in the first step, i.e. the higher heating value HHV.

#### The combustion inside IFCS

The mass balance for IFCS considers chemical oxidation reactions without dissociation of the gaseous fuel. The mass balance allowed the computation of inlet and outlet mole fractions of all chemical species. The Table 4 includes the mole fractions in the flow section “0” and the Table 5 includes the mole fractions in the flow section “1”.

**Table 5:** The mole composition in the flow section ”1”.

y_CO2_ [kmole]	z_H2O_ [kmole]	r_O2_ [kmole]	r_N2_ [kmole]
0.89706	0.762916	1.5795^.^x	5.9418^.^(1 + x)

Where y_CO2_ = n_CO_ + n_CH4_ + 2 n_C2H4_ + 2 n_C2H6_ + 3 n_C3H6_ + 3 n_C3H8_ + 4 n_C4H10_.

z_H2O_ = 0.5 n_H2_ + n_CH4_ + n_C2H4_ + 1.5 n_C2H6_ + 1.5 n_C3H6_ + 2 n_C3H8_ + 2.5 n_C4H10_.

The energy balance for IFCS computed the value of the combustion higher heating value, HHV, released by the constant temperature and constant pressure combustion of the fuel.

$$\sum\limits_{outlet} {N_{i} \cdot h_{f,i}^{0} } - \sum\limits_{inlet} {N_{j} \cdot h_{f,j}^{0} } = HHV\quad \left[ {\text{kJ/kmole}} \right]$$

*N*_*i*_ and $h_{f,i}^{0}$ are the kmole number and the standard enthalpy of formation for each outlet chemical species, *N*_*j*_ and $h_{f,j}^{0}$ are the kmole number and the standard enthalpy of formation for each inlet chemical species. Thus it is yielding, see Table 6:

1 $$\begin{aligned} HHV & = y_{CO2} \cdot h_{f,CO2}^{0} + z_{H2O} \cdot h_{f,H2O}^{0} - n_{CO} \cdot h_{f,CO}^{0} - n_{CH4} \cdot h_{f,CH4}^{0} - n_{C2H4} \cdot h_{f,C2H4}^{0} \\ & - n_{C2H6} \cdot h_{f,C2H6}^{0} - n_{C3H6} \cdot h_{f,C3H6}^{0} - n_{C3H8} \cdot h_{f,C3H8}^{0} - n_{C4H10} \cdot h_{f,C4H10}^{0} = - 5.15403 \cdot 10^{5} \quad \left[ {\text{kJ/kmole}} \right] \\ \end{aligned}$$

**Table 6:** The enthalpy of formation of chemical species in the flow sections “0”, “1”.

${h}_{f,O2}^{0}$ [kJ/kmole]	${h}_{f,N2}^{0}$ [kJ/kmole]	${h}_{f,H2}^{0}$ [kJ/kmole]	${h}_{f,CO}^{0}$ [kJ/kmole]	${h}_{f,CO2}^{0}$ [kJ/kmole]	${h}_{f,CH4}^{0}$ [kJ/kmole]	${h}_{f,C2H4}^{0}$ [kJ/kmole]	${h}_{f,C2H6}^{0}$ [kJ/kmole]	${h}_{f,C3H6}^{0}$ [kJ/kmole]	${h}_{f,C3H8}^{0}$ [kJ/kmole]	${h}_{f,C4H10}^{0}$ [kJ/kmole]	${h}_{f,H2O}^{0}$ [kJ/kmole]
0	0	0	− 110,527	− 393,522	− 74,873	+ 52,467	− 84,740	+ 20,430	− 103,900	− 126,800	− 285,830

p_0_ = p_1_ = 0.8 MPa, T_0_ = T_1_ = 298 K^38–44^.

#### The flue gases heating with dissociation inside FHDS

The non-dissociated flue gases leaving the IFCS enter the FHDS where two simultaneous processes take place:

- chemical reactions of dissociation and recombination controlled by chemical equilibrium constants and
- constant pressure heating of all chemical species, non or dissociated ones.

**Figure 12:**
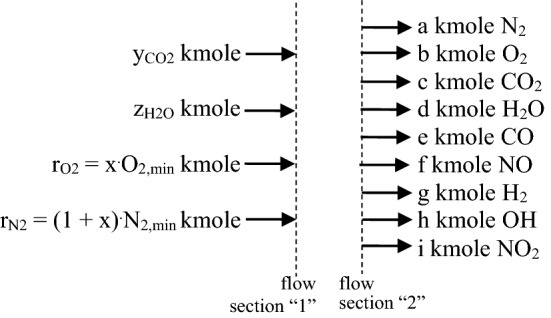
The inlet/outlet flow scheme of chemical species for FHDS.

The both processes are completed by consuming the HHV. The flow scheme required by the mass and energy balance laws is presented in the Fig. 12. The below chemical reactions of dissociation and recombination were considerated.

2 $$2^{.} {\text{CO}}_{{2}} \to 2^{.} {\text{CO}} + {\text{O}}_{{2}}$$

3 $$2^{.} {\text{H}}_{{2}} {\text{O}} \to 2^{.} {\text{H}}_{{2}} + {\text{O}}_{{2}}$$

4 $$2^{.} {\text{H}}_{{2}} {\text{O}} \to 2^{.} {\text{OH}} + {\text{H}}_{{2}}$$

5 $${\text{N}}_{2} + {\text{O}}_{2} \to {\text{2NO}}$$

6 $${\text{N}}_{{2}} + \, 2^{.} {\text{O}}_{{2}} \to 2^{.} {\text{NO}}_{2}$$

The mass balance for FHDS gives the following four dependences:

7 $${\text{a}} = {\text{r}}_{{{\text{N2}}}} {-} \, 0.5^{.} {\text{f}}{-}0.5^{.} {\text{i}}$$

8 $${\text{b}}: = {\text{r}}_{{{\text{O2}}}} {-} \, 0.5^{.} {\text{f }}{-}{\text{ i}} + 0.5^{.} {\text{e }} + \, 0.5^{.} {\text{g}}$$

9 $${\text{c}} = {\text{y}}_{{{\text{CO2}}}} {-}{\text{e}}$$

10 $${\text{d}} = {\text{z}}_{{{\text{H2O}}}} {-}{\text{g}}{-}{\text{h}}$$

The mass balance is completed trough chemical equilibrium constants K.

$$\nu_{{\text{A}}} \cdot {\text{A }} + \nu_{{\text{B}}} \cdot {\text{B}} \to \nu_{{\text{C}}} \cdot {\text{C }} + \nu_{{\text{D}}} \cdot {\text{D}} \Rightarrow K = \frac{{C^{{\nu_{C} }} \cdot D^{{\nu_{D} }} }}{{A^{{\nu_{A} }} \cdot B^{{\nu_{B} }} }} \cdot \left( {\frac{p}{{p_{0} }}} \right)^{{\nu_{C} + \nu_{D} - \nu_{A} - \nu_{B} }}$$

Therefore they are yielding:

- dissociation of CO_2_
  11 $$K1 = \frac{{e^{2} \cdot b}}{{c^{2} }} \cdot \left( {\frac{p}{{p_{0} }}} \right)^{2 + 1 - 2} = \frac{{e^{2} \cdot b}}{{c^{2} }} \cdot \left( {\frac{p}{{p_{0} }}} \right) \Rightarrow e = \left( {\frac{{K1 \cdot c^{2} }}{b} \cdot \frac{{p_{0} }}{p}} \right)^{0.5}$$
- dissociation of H_2_O giving OH and H_2_
  12 $$K2 = \frac{{g^{2} \cdot b}}{{d^{2} }} \cdot \left( {\frac{p}{{p_{0} }}} \right)^{2 + 1 - 2} = \frac{{g^{2} \cdot b}}{{d^{2} }} \cdot \left( {\frac{p}{{p_{0} }}} \right) \Rightarrow g = \left( {\frac{{K2 \cdot d^{2} \cdot p_{0} }}{b \cdot p}} \right)^{0.5}$$
- dissociation of H_2_O giving H_2_ and O_2_
  13 $$K3 = \frac{{h^{2} \cdot g}}{{d^{2} }} \cdot \left( {\frac{p}{{p_{0} }}} \right)^{2 + 1 - 2} = \frac{{h^{2} \cdot g}}{{d^{2} }} \cdot \left( {\frac{p}{{p_{0} }}} \right) \Rightarrow h = \left( {\frac{{K3 \cdot d^{2} \cdot p_{0} }}{g \cdot p}} \right)^{0.5}$$
- recombination of N_2_ and O_2_ giving NO
  14 $$K4 = \frac{{f^{2} }}{a \cdot b} \cdot \left( {\frac{p}{{p_{0} }}} \right)^{2 - 1 - 1} \Rightarrow f = \left( {K4 \cdot a \cdot b} \right)^{0.5}$$
- recombination of N_2_ and O_2_ giving NO_2_
  15 $$K5 = \frac{{i^{2} }}{{a \cdot b^{2} }} \cdot \left( {\frac{p}{{p_{0} }}} \right)^{2 - 1 - 2} = \frac{{i^{2} }}{{a \cdot b^{2} }} \cdot \left( {\frac{{p_{0} }}{p}} \right) \Rightarrow i = \left( {\frac{{K5 \cdot a \cdot b^{2} \cdot p}}{{p_{0} }}} \right)^{0.5}$$

The Eqs. (7 to 15) could be solved if there are known the flue gases temperature, T_fg_, in the flow section “2” and the excess air, x, in the flow section “1”.

The energy balance gives the extra dependence between unknown mole composition of flue gases leaving FHDS and T_fg_ and x.

16 $$\begin{aligned} & a \cdot \int_{{T_{0} }}^{{T_{fg} }} {c_{p,N2} } \cdot dT + b \cdot \int_{{T_{0} }}^{{T_{fg} }} {c_{p,O2} } \cdot dT + c \cdot \left( {h_{f,CO2}^{0} + \int_{{T_{0} }}^{{T_{fg} }} {c_{p,CO2} } \cdot dT} \right) + d \cdot \left( {h_{f,H2O}^{0} + \int_{{T_{0} }}^{{T_{fg} }} {c_{p,H2O} } \cdot dT} \right) \\ & \quad + e \cdot \left( {h_{f,CO}^{0} + \int_{{T_{0} }}^{{T_{fg} }} {c_{p,CO} } \cdot dT} \right) + f \cdot \left( {h_{f,NO}^{0} + \int_{{T_{0} }}^{{T_{fg} }} {c_{p,NO} } \cdot dT} \right) + \left( {g + 0.5 \cdot h} \right) \cdot \int_{{T_{0} }}^{{T_{fg} }} {c_{p,H2} } \cdot dT \\ & \quad + h \cdot \left( {h_{f,OH}^{0} + \int_{{T_{0} }}^{{T_{fg} }} {c_{p,OH} } \cdot dT} \right) + i \cdot \left( {h_{f,NO2}^{0} + \int_{{T_{0} }}^{{T_{fg} }} {c_{p,NO2} } \cdot dT} \right) - y_{CO2} \cdot h_{f,CO2}^{0} - z_{H2O} \cdot h_{f,H2O}^{0} = \left| {HHV} \right| \\ \end{aligned}$$

The specific heat capacities, c_p_, were approximated by polynomial dependences on temperature. The enthalpy of formation of new chemical species is included in the Table 7.

**Table 7:** The enthalpy of formation of chemical species in the flow sections “2”.

${h}_{f,O2}^{0}$ [kJ/kmole]	${h}_{f,N2}^{0}$ [kJ/kmole]	${h}_{f,H2}^{0}$ [kJ/kmole]	${h}_{f,CO}^{0}$ [kJ/kmole]	${h}_{f,CO2}^{0}$ [kJ/kmole]	${h}_{f,NO}^{0}$ [kJ/kmole]	${h}_{f,NO2}^{0}$ [kJ/kmole]	${h}_{f,OH}^{0}$ [kJ/kmole]	${h}_{f,H2O}^{0}$ [kJ/kmole]
0	0	0	− 110,527	− 393,522	+ 90,291	+ 33,100	+ 38,897	− 285,830

p_0_ = p_1_ = p_2_ = 0.8 MPa, T_0_ = T_1_ = 298 K, T_2_ = T_fg_, ^37–43^.

Because the Eqs. (7 to 15) includes large quantities, i.e. a, b, c, d, and small, very small or negligible quantities, i.e. e, f, g, h, i, the computational procedure starts with Eqs. (11 to 16) in which the temperature T_fg_ was imposed and supposed that a ≃ r_N2_, b ≃ r_O2_, c ≃ y_CO2_ and d ≃ z_H2O_. The preliminary evaluation of e, f, g, h and i allows the first evaluation of a, b, c and d through Eqs. (7 to 10) followed by a preliminary evaluation of excess air through Eq. (16). Knowing the first values of all chemical species and x, the next step re-uses the Eqs. (7 to 15) and after those Eqs. (11 to 15, 16). The iterative procedure is finished when the imposed error between two successive iterations is reached. The numerical results are included in the Tables 8 and 9.

**Table 8:** The mole composition of flue gases function of temperature T_fg_, flow section “2”.

x [kmole/kmole fuel]	T_fg_ [K]	e_CO_ [kmole/kmole fuel]	f_NO_ [kmole/kmole fuel]	g_H2_ [kmole/kmole fuel]	h_OH_ [kmole/kmole fuel]	i_NO2_ [kmole/kmole fuel]
10.425	500	7.417*10^−27^	5.505*10^−8^	1.937*10^−25^	4.548*10^−15^	1.0211*10^−5^
2.0625	1000	1.0657*10^−11^	6.665*10^−4^	1.3063*10^−11^	3.907*10^−7^	4.88*10^−5^
1.3175	1200	3.822*10^−9^	2.841*10^−3^	2.376*10^−9^	9.031*10^−6^	5.16*10^−5^
0.855	1400	2.668*10^−7^	7.469*10^−3^	1.049*10^−7^	8.295*10^−5^	4.7472*10^−5^
0.547	1600	6.796*10^−6^	1.4379*10^−2^	1.9308*10^−6^	4.243*10^−4^	3.914*10^−5^
0.3345	1800	9.024*10^−5^	2.211*10^−2^	2.0188*10^−5^	1.453*10^−3^	2.891*10^−5^
0.1835	2000	1.226*10^−4^	1.531*10^−2^	2.746*10^−5^	1.245*10^−3^	1.471*10^−5^
0.0765	2200	5.607*10^−3^	2.756*10^−2^	1.07*10^−3^	7.654*10^−3^	8.304*10^−6^

p_2_ = 0.8 MPa.

**Table 9:** The mole composition of flue gases function of temperature T_fg_, flow section “2”.

x [kmole/kmole fuel]	T_fg_ [K]	e_CO_ [kmole/kmole fuel]	f_NO_ [kmole/kmole fuel]	g_H2_ [kmole/kmole fuel]	h_OH_ [kmole/kmole fuel]	i_NO2_ [kmole/kmole fuel]
10.425	500	2.098*10^−26^	5.505*10^−8^	5.479*10^−25^	7.649*10^−15^	2.888*10^−5^
2.0625	1000	3.014*10^−11^	6.665*10^−4^	3.694*10^−11^	6.571*10^−7^	1.38*10^−4^
1.3175	1200	1.081*10^−8^	2.840*10^−3^	6.720*10^−9^	1.518*10^−5^	1.459*10^−4^
0.855	1400	7.547*10^−7^	7.47*10^−3^	2.967*10^−7^	1.395*10^−4^	1.343*10^−4^
0.5472	1600	1.922*10^−5^	1.438*10^−2^	5.461*10^−6^	7.136*10^−4^	1.107*10^−4^
0.3335	1800	2.557*10^−4^	2.205*10^−2^	5.704*10^−5^	2.437*10^−3^	8.136*10^−5^
0.1825	2000	3.461*10^−4^	1.532*10^−2^	7.756*10^−5^	2.095*10^−3^	4.168*10^−5^
0.0670	2200	1.652*10^−2^	2.636*10^−2^	2.679*10^−3^	1.162*10^−2^	2.157*10^−5^

p_2_ = 0.1 MPa.

Tables 8 and 9 present the main combustion noxious emissions, i.e. CO, NO and NO_2_ calculated using the chemical modeling algorithm at different temperatures, pressure and excess of air/oxygen. The emission levels calculated using the model had in most cases the same variation trend. For example, at high excess of air/oxygen and low temperature the emission of CO, NO and NO_2_ calculated have the minimum values. This result was expected, as the combustion process was performed under conditions of high oxygen excess that maximize the formation of complete combustion products. Opposite, at low air/oxygen excess and high temperature, the combustion noxious emission presents maximum values, but in most cases, above the legal limit. However, emissions under hydrotreating conditions should not be compared with proposals within legal limits, since these limits refer mainly to combustion conditions with excess air ^44^. The gaseous fraction resulted from hydrotreating experiments contains a high amount of unreacted hydrogen. In conclusion, the developed model allows the evaluation of harmful emissions depending on the temperature and pressure set on the flue gases. This calculation algorithm can be applied to any pressurized or atmospheric combustion engine fueled with different mixture of combustible gases such as: natural gas, hydrogen-enriched natural gas or oxy-fuel.

## Conclusions

CoMo/γ-Al_2_O_3_-HMS catalyst was tested in the hydrotreating process of pyrolysis bio-oil. Based on the presented results at mild conditions of 320 °C and 40 bar, the catalyst is very active in the hydrotreating process leading to a decrease of total acid number of hydrotreated bio-oil with almost 89% and bio-oil conversion of 87.23%. In addition, in order to evaluate the harmful emissions resulted from combustion of gaseous phase obtained in the hydrotreating process a chemical modeling algorithm was developed**.**

## Methods

### Catalyst characterization

The surface area, pore volume and mean pore diameter of the catalyst width was calculated by the Brunauer-Emmet-Teller (BET) method. The nitrogen sorption measurements were carried out at 77 K using a Nova 1000 Quantachrome instrument. Prior to analysis, the samples were heated to 200 °C for 1 h. The X-ray diffraction (XRD) analysis was carried out using a fully automated, modular Rigaku Smart Labdiffractometer, operated at 45 kV and 200 mA, with Cu Kα radiation (1.54059 Ǻ), parallel beam configuration (2θ/θ scan mode) and scanning range between 2–90° (2θ), with a 0.02° step. Fourier transform infrared (FT-IR) analysis was performed with a FT-IR Tensor 27—Bruker spectrometer, using KBr pellet technique ^45^. Scanning Electron Microscopy (SEM) analysis was conducted using a FEI Inspect, S model microscope. The X-Ray Photoelectron Spectroscopy (XPS) spectra were obtained on a SPECS spectrometer by using the Al anode (1486.6 eV) radiation. The pyridine-adsorbed FT-IR spectrum was recorded using Jasco 610 spectrometer, with a scanning range from 4000 to 400 cm^−1^, a scan rate of 4 cm^-1^·s^-1^ and an average of 64 measurements in the final spectrum. The total number of acidic sites (sites/m2) was measured by temperature programmed desorption of pyridine (TPD-pyridine) as described by using a TGA Q5000 v3.13 from TA Instruments, USA For the TPD-pyridine profile as described by using TGA Q5000 v3.13 from TA Instruments, USA.

## Experimental part

### Catalyst preparation

The HMS material was synthesized via templating pathway using tetraethylorthosilicate (TEOS) as silica source and dodecylamine (DDA, Merck) as template and ethanol (EtOH) and water as solvents. Tetraethylorthosilicate (TEOS) was added to a mixture of dodecylamine (DDA), water and ethanol at room temperature. The mixture was stirred for 1 h at 40 °C and then let for 24 h at 25 °C. The molar composition of the material was 1.0 SiO_2_: 0.27 DDA: 8.5 EtOH: 29 H_2_O. The solid was filtered, washed, dried at room temperature and calcined in air at 550 °C for 8 h (1 °C/min), to remove the template. The γ-Al_2_O_3_-HMS support was obtained as cylindrical extrudates with the average size of 2 mm. The powdered materials (γ-Al_2_O_3_/HMS: 40/60) were mixed with a 10% (wt%) HNO_3_ solution gradually dosed for 1 h. After dosing the nitric acid, stirring is continued for 3 h at ambient temperature. The resulted homogeneous paste is inserted into a manually operated extruder with interchangeable diameters, the diameter of a die having the size of 1 mm. The resulted catalyst were dried for 6 h at 160 °C and annealed at 450 °C (10 °C/min). The molybdenum (8%) and cobalt (4%) catalyst was prepared by incipient wetness impregnation method using appropriate concentrations of aqueous solutions of ammonium heptamolybdate ((NH_4_)_6_Mo_7_O_24_, Aldrich 99%) and cobalt (II) nitrate hexahydrate (Co(NO_3_)_2_·6H_2_O), Aldrich 98%). After impregnation on γ-Al_2_O_3_-HMS support the wet cylinders were dried at 120 °C for 4 h and calcined at 450 °C for 2 h.

### Catalytic tests

The activity tests of 4%Co8%Mo/ɤ-Al_2_O_3_-HMS catalyst were carried out in a fixed bed flow reactor (length of 0.5 m, volume of 200 cm^3^) heated by an electrical furnace. An amount of 50 cm^3^ catalyst was loaded in the middle zone of the reactor. The catalyst was activated in situ with a flow rate of 15 L/h hydrogen at 450 °C for 6 h. The bio-oil used in this report was obtained by pyrolysis of biomass derivate from biogas process and conditioned with waste vegetable oil at 425 °C ^46^. Bio-oil was introduced into the reactor using a metering pump, mixed with H_2_, and preheated to a desired temperature. The liquid samples were analyzed by GC/MS 7000 Triple Quad MS (Agilent Technologies) system equipped with HP-FFAP (30 m, 250 μm, 0.25 μm) column and He as carrier gas with volumetric flow of 1 ml/min. The oven program started from 30 °C with 2 °C/min rate and until reached 100 °C. The injector temperature was set at 250 °C. The compounds were identified using NIST MS Search 2.0 Library. The water content in the samples was determined by Karl Fischer method (ASTM D6869). Density, total acid number and saponification number were determined using standardized methods ^47^.

The gaseous fraction generated during the experiments was analyzed using a Agilent Technologies 6890 N Gas-Chromatograph with a Thermal Conductivity Detector (TCD), connected on-line, equipped with a SHINCARBON ST 80/100 2 M, 2 M ID,1/8"OD, SILCO, HP column and He as carrier gas with volumetric flow of 50 mL/min. The column temperature was set up to 40 °C (hold time) 300 °C, program rate: 20 °C/min, injection temperature: 300 °C, detector temperature 300 °C, sample volume: 0.75 mL.

The product yields in were calculated using the following equations:

$${\eta }_{i}(\%)=\frac{\frac{{x}_{i}\cdot {\overline{M}}_{oil}}{{M}_{i}}}{(\sum_{i=1}^{n}\frac{{x}_{i}\cdot {\overline{M}}_{oil}}{{M}_{i}})+{x}_{unreacted}}\cdot 100$$

where, M_*oil*_ -average molecular weight of the bio-oil, X_*i*_-mass fraction of the component I, M_*i*_-molecular weight of component i

$$Conversion(\%)=\sum_{i=1}^{n}{\eta }_{i}(\%)$$

## Supplementary Information

Below is the link to the electronic supplementary material.

Supplementary Information 1.

## References

[1] Wiśniewski D, Gołaszewski J, Białowiec A (2015). The pyrolysis and gasification of digestate from agricultural biogas plant. Arch. Environ. Prot..

[2] Hosseini Koupaie E, Azizi A, Bazyar Lakeh AA, Hafez H, Elbeshbishy E (2019). Comparison of liquid and dewatered digestate as inoculum for anaerobic digestion of organic solid wastes. Waste Manag..

[3] Jung-Hun K, Jeong-Ik O, Yiu Fai T, Young-Kwon P, Jechan L, Eilhann EK (2020). CO2-assisted catalytic pyrolysis of digestate with steel slag. Energy.

[4] Horácˇek J, Kubicˇka D (2017). Bio-oil hydrotreating over conventional CoMo&NiMo catalysts: The role of reaction conditions and additives. Fuel.

[5] Vituruch G, Boonyawan Y, Tanakorn R, Sabaithip T (2015). Hydrotreating of free fatty acid and bio-oil model compounds: effect of catalyst support. Energy Proc..

[6] Botella L, Stankovikj F, Sánchez JL, Gonzalo A, Arauzo J, Garcia-Pérez M (2018). Bio-oil hydrotreatment for enhancing solubility in biodiesel and the oxydation stability of resulting blends. Front. Chem..

[7] Wang H, Meyer PA, Santosa DM, Zhu C, Olarte MV, Jones SB, Zacher AH (2020). Performance and techno-economic evaluations of co-processing residual heavy fraction in bio-oil hydrotreating.

[8] French RJ, Hrdlicka J, Baldwina R (2010). Mild hydrotreating of biomass pyrolysis oils to produce a suitable refinery feedstock. Environ. Prog. Sustain..

[9] Wildschut J, Melián-Cabrera I, Heeres HJ (2010). Catalyst studies on the hydrotreatment of fast pyrolysis oil. Appl. Catal. B-Environ..

[10] Cai Q, Yu T, Meng X, Zhang S (2020). Selective generation of aromatic hydrocarbons from hydrotreating-cracking of bio-oil light fraction with MOx modified HZSM-5 (M = Ga, Mo and Zn). Fuel Process..

[11] Wildschut J, Mahfud FH, Venderbosch RH, Heeres HJ (2009). Hydrotreatment of fast pyrolysis oil using heterogeneous noble-metal catalysts. Ind. Eng. Chem. Res..

[12] Mendes, F.L., Teixeira da Silva, V., Pacheco, M.E., Toniolo, F.S., Henriques, C.A. Bio-oil hydrotreating using nickel phosphides supported on carbon-covered alumina. Fuel. **241**, 686–694, (2019).

[13] Jin W, Pastor-Pérez L, Shen D, Sepúlveda-Escribano A, Gu S, Reina TR (2019). Catalytic upgrading of biomass model compounds: novel approaches and lessons learnt from traditional hydrodeoxygenation: a review. ChemCatChem.

[14] Zhong-Yu, J., Zhang, T.-Q., Shang, J.-W., Zhai, M.-Lu, Yang, H., Qiao, C.-Z., Ma X.-Q. Influence of Cu and Mo components of ɤ-Al2O3 supported nickel catalysts on hydrodeoxygenation of fatty acidmethyl esters to fuel-like hydrocarbons. J. Fuel Chem. Technol. **46(4)**, 427–440, (2018).

[15] Ranga C, Alexiadis VI, Lauwaert J, Lødeng R, Thybaut JW (2019). Effect of Co incorporation and support selection on deoxygenation selectivity and stability of (Co)Mo catalysts in anisole HDO. Appl. Catal. A Gener..

[16] Schmitt CC, Raffelt K, Zimina A, Krause B, Otto T, Rapp M, Grunwaldt J-D, Dahmen N (2018). Hydrotreatment of fast pyrolysis bio-oil fractions over nickel-based catalyst. Top. Catal..

[17] Mora-Vergara D, Moscoso LH, Gaigneaux EM, Giraldo SA, Baldovino-Medrano VG (2018). Hydrodeoxygenation of guaiacol using NiMo and CoMo catalysts supported on alumina modified with potassium. Catal. Today..

[18] Prasomsri T, Shetty M, Murugappan K, Román-Leshkov Y (2014). Insights into the catalytic activity and surface modification of MoO3 during the hydrodeoxygenation of lignin-derived model compounds into aromatic hydrocarbons under low hydrogen pressures. Energy Environ. Sci..

[19] Li Y, Zhang C, Liu Y, Hou X, Zhang R, Tang X (2015). Coke deposition on Ni/HZSM-5 in bio-oil hydrodeoxygenation processing. Energy Fuels..

[20] Kadarwati S, Hu X, Gunawan R, Westerhof R, Gholizadeh M, Mahmudul Hasan MD, Li C-Z (2017). Coke formation during the hydrotreatment of bio-oil using NiMo and CoMo catalysts. Fuel Process. Technol..

[21] Yoosuk B, Sanggam P, Wiengket S, Prasassarakich P (2019). Hydrodeoxygenation of oleic acid and palmitic acid to hydrocarbon-like biofuel over unsupported Ni-Mo and Co-Mo sulfide catalysts. Renew. Energy..

[22] Ropero-Vega, J.L., Aldana-Pérez, A., Gómez, R., Ni˜no-Gómez, M.E. Sulfated titania [TiO2/SO42−]: a very active solid acid catalyst for the esterification of free fatty acids with ethanol. Appl. Catal. A Gener. **379(1–2),** 24–29, (2010).

[23] Palcheva R, Kaluza L, Dimitrov L, Tyuliev G, Avdeev G, Jiratova K, Spojakina A (2016). NiMo catalysts supported on the Nb modified mesoporous SBA-15 and HMS: effect of thioglycolic acid addition on HDS. Appl. Catal. A: Gen..

[24] Yazici D. T., Bilgic, C. Surf. Interface Anal. Determining the surface acidic propertiesof solid catalysts by amine titration usingHammett indicators and FTIR-pyridineadsorption methods. **42**, 959–962, (2010).

[25] Barzetti T, Selli E, Moscotti D, Forni L (1996). Pyridine and ammonia as probes for FTIR analysis of solid acid catalysts. J. Chem. Soc. Faraday Trans..

[26] Osman AI, Abu-Dahrieh JK, Rooney DW, Halawy SA, Mohamed MA, Abdelkader A (2012). Effect of precursor on the performance of alumina for the dehydration of methanol to dimethyl ether. Appl. Catal. B Environ..

[27] NIST Standard Reference Database 20, Version 4.1. https://srdata.nist.gov/xps/.

[28] Zepeda, T.A, Pawelec, B., Obeso-Estrella, R., Díaz de León, J.N, Fuentes S, Alonso-Núñez, G., et al. Competitive HDS and HDN reactions over NiMoS/HMS-Al catalysts: Diminishing of the inhibition of HDS reaction by support modification with P. Appl. Catal. B: Environ. **180**, 569–579 (2016).

[29] Ganta D, Sinha S, Haasch RT (2014). 2-D material molybdenum disulfide analyzed by XPS. Surf. Sci. Spectra.

[30] Yang J, Liu H, Martens WN, Frost RL (2010). Synthesis and characterization of cobalt hydroxide, cobalt oxyhydroxide, and cobalt oxide nanodiscs. J. Phys. Chem. C..

[31] Turner NH, Single AM (1990). Determination of peak positions and areas from wide-scan XPS spectra. Surf. Interface Anal..

[32] Zafeiratos S, Dintzer T, Teschner D, Blume R, Hävecker M, Knop-Gericke A (2010). Methanol oxidation over model cobalt catalysts: Influence of the cobalt oxidation state on the reactivity. J. Catal..

[33] Mosallanejad A, Taghvaei H, Mirsoleimani-Azizi SM, Mohammadi A, Rahimpour MR (2017). Chem. Eng. Res. Des..

[34] Zerva, C., Karakoulia, S.A., Kalogiannis, K.G., Margellou, A., Iliopoulou, E.F., Lappas, A.A., Papayannakos, N, Triantafyllidis, K.S. Hydrodeoxygenation of phenol and biomass fast pyrolysis oil (bio-oil) over Ni/WO3-ZrO2 catalyst. Catal. Today. 2020, in Press.

[35] Oh S, Lee JH, Choi JW (2020). Hydrodeoxygenation of crude bio-oil with various metal catalysts in a continuous-flow reactor and evaluation of emulsion properties of upgraded bio-oil with petroleum fuel. Renew. Energy..

[36] Yang Y, Wang Q, Zhang X, Wang L, Li G (2013). Hydrotreating of C18 fatty acids to hydrocarbons on sulphided NiW/SiO2-Al2O3. Fuel Process. Technol..

[37] Borgnakke, C., Sonntag , R.E., Fundamentals of Thermodynamics, 8th Edition, John Wiley & Sons, 2013.

[38] Ruscic B, Pinzon RE, Morton ML, von Laszewski G, Bittner S, Nijsure SG, Amin KA, Minkoff M, Wagner AF (2004). Introduction to active thermochemical tables: several "key" enthalpies of formation revisited. J. Phys. Chem. A.

[39] Ruscic B, Pinzon RE, von Laszewski G, Kodeboyina D, Burcat A, Leahy D, Montoya D, Wagner AF (2005). Active thermochemical tables: thermochemistry for the 21st century. J. Phys. Conf. Ser..

[40] Ruscic, B. Active Thermochemical Tables (ATcT) values based on ver. 1.118 of the Thermochemical Network (2015); available at ATcT.anl.gov

[41] Ruscic, B. Active thermochemical tables: dissociation energies of several homonuclear first-row diatomics and related thermochemical values. Theor. Chem. Acc. **133**, 1415/1–12 (2005)

[42] Klippenstein, S. J., Harding, L. B., B. Ruscic, Ab initio computations and active thermochemical tables hand in hand: heats of formation of core combustion species. J. Phys. Chem. A **121**, 35, 6580–6602 (2017). 10.1021/acs.jpca.7b05945 28758403

[43] Horbaniuc B, Marin O, Dumitrascu G, Charon O (2004). Oxygen enriched combustion in supercritical steam boilers. Energy.

[44] Conesa JA, Ortuño N, Palmer D (2020). Estimation of industrial emissions during pyrolysis and combustion of different wastes using laboratory data. Sci Rep.

[45] Enascuta C-E, Stepan E, Bolocan I, Bombos D, Calin C, Oprescu E-E, Lavric V (2018). Simultaneous production of oil enriched in ω-3 polyunsaturated fatty acids and biodiesel from fish wastes. J. Waste Manag..

[46] Doukeh, R., Bombos, M., Bombos,D., Vasilievici, G., Radu E., Oprescu, E-E. Pyrolysis of digestate from anaerobic digestion on tungsten oxide catalyst. Reaction Kinetics, Mechanisms and Catalysis. 2021, in press. 10.1007/s11144-021-01952-7.

[47] Iisa K, French RJ, Orton KA, Dutta A, Schaidle JA (2017). Production of low-oxygen bio-oil via ex situ catalytic fast pyrolysis and hydrotreating. Fuel.

