# Supplementary material for: Catalytic hydrotreating of bio-oil and evaluation of main noxious emissions of gaseous phase
Source: Sci Rep. 2021 Mar 17;11:6176. doi: 10.1038/s41598-021-85244-z (PMC7969750; doi:10.1038/s41598-021-85244-z)
Supplement: Supplementary file 1 — Supplementary Information 1. [file 41598_2021_85244_MOESM1_ESM.docx]

**Supplementary material**

**Catalytic hydrotreating of bio-oil and evaluation of main noxious emissions of gaseous phase**

**Rami Doukeh^1^, Dorin Bombos^1^, Mihaela Bombos^2^, Elena-Emilia Oprescu^1,2^, Gheorghe Dumitrascu^3^¸ Vasilievici Gabriel^2^, Catalina Calin^1*^**

^1^Chemistry Department, Petroleum-Gas University of Ploiesti, 39 Bucuresti Blvd., 100680, Ploiesti, Romania

^2^National Institute for Research and Development for Chemistry and Petrochemistry ICECHIM Bucuresti, 202 Splaiul Independentei, 060021, Bucharest, Romania

^3^Department of Engineering Thermodynamics, ‘‘Gh. Asachi’’ Technical University of Iasi, Bd. D. Mangeron, 59-61,6600 Iasi, Romania

*Correspondence to:* [*catalina.calin20@yahoo.com*](mailto:catalina.calin20@yahoo.com)*; catalina.calin@upg-ploiesti.ro;* [*oprescuemilia@gmail.com*](mailto:oprescuemilia@gmail.com)

*Number of pages: 2*

*Number of figures: 1*

*Number of tables : 1*

Table S1. Chemical composition of pyrolysis bio-oil

| Peak Number | Retention time | Area Sum % |
| --- | --- | --- |
| Ethyl levulinate | 5.903 | 3.24 |
| Phenol, 4-methyl | 6.103 | 0.85 |
| Phenol, 2-methoxy | 6.286 | 0.88 |
| Tetradecane | 10.531 | 0.96 |
| Naphtalene, 1,3-dimethyl | 11.002 | 1.5 |
| Benzil alcohol, α-isobutyl, 3,4,6-trimethyl | 11.816 | 5.89 |
| Naphtalene, 2,3,6-trimethyl | 12.789 | 6.8 |
| Hexadecane | 13.044 | 7.97 |
| Naphtalene, 1,6,7-trimethyl | 13.116 | 3.29 |
| Tetradecane, 2,6,10-trimethyl | 13.571 | 4.22 |
| Heptadecan | 14.217 | 19.22 |
| Octadecane | 15.31 | 11.92 |
| Octadecane, 3-ethyl-5-(2-ethylbutyl) | 15.366 | 3.97 |
| Nonadecane | 16.356 | 11.13 |
| Heneicosane | 17.337 | 3.52 |
| Heptacosane | 18.295 | 3.71 |
| 1-hexadecanol, 2 -mehtyl | 19.204 | 0.73 |
| Other small compounds | - | 10.20 |


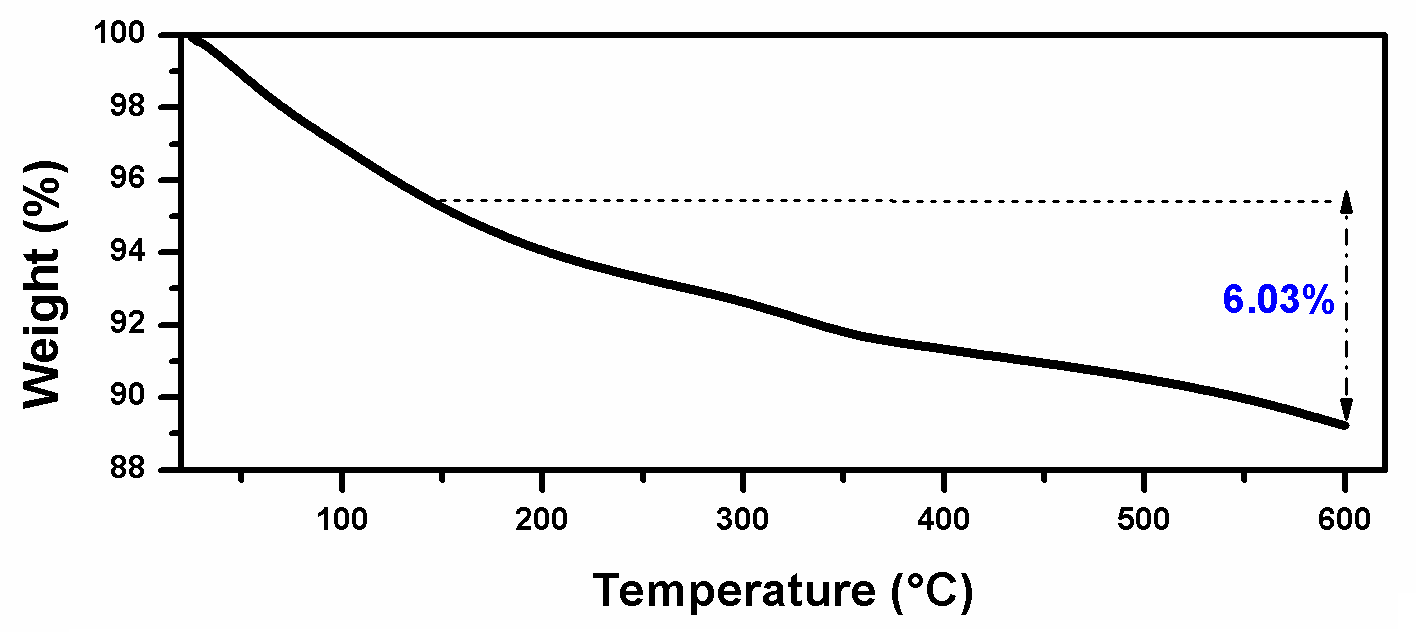


Figure S1. TPD-pyridine profile of the CoMo/γ-Al_2_O_3_-HMS catalyst
